# Comparison of Diagnostic Performance of Whole-Body 360° CZT Bone SPECT/CT and Planar Bone Scintigraphy in Detecting Bone Metastasis in Small Cell Lung Cancer

**DOI:** 10.3390/diagnostics16172838

**Published:** 2026-09-03

**Authors:** Sang Mi Lee, Su Jin Jang, Ju Ock Na, Ki Hyun Seo, Nam Hun Heo, Jeong Won Lee

**Affiliations:** 1 Department of Nuclear Medicine, Soonchunhyang University Cheonan Hospital, 31 Suncheonhyang 6-gil, Dongnam-gu, Cheonan 31151, Republic of Korea; 2 Department of Nuclear Medicine, CHA Bundang Medical Center, CHA University School of Medicine, 59 Yatap-ro, Bundang-gu, Seongnam 13496, Republic of Korea; 3 Department of Internal Medicine, Soonchunhyang University Cheonan Hospital, 31 Suncheonhyang 6-gil, Dongnam-gu, Cheonan 31151, Republic of Korea; 4 Clinical Trial Center, Soonchunhyang University Cheonan Hospital, 31 Suncheonhyang 6-gil, Dongnam-gu, Cheonan 31151, Republic of Korea

**Keywords:** cadmium zinc telluride, bone metastasis, bone scintigraphy, single photon emission computed tomography, small cell lung cancer

## Abstract

**Background/Objectives**: We aimed to compare the diagnostic performances of planar bone scintigraphy and whole-body 360° cadmium–zinc–telluride (CZT) bone single-photon emission computed tomography/computed tomography (SPECT/CT) in detecting bone metastasis in patients with small cell lung cancer (SCLC). **Methods**: We retrospectively evaluated two separate, non-randomized cohorts of 175 patients with SCLC who underwent staging bone scintigraphy, comprising 93 and 82 patients who underwent planar bone scintigraphy using a conventional dual-head gamma camera and whole-body bone SPECT/CT using a full-ring 360° CZT camera, respectively. Both imaging examinations were visually assessed and compared using patient- and lesion-based analyses of their diagnostic performances. **Results**: Whole-body bone SPECT/CT showed fewer equivocal findings and almost perfect inter-reader agreement. On the patient-based analysis, whole-body bone SPECT/CT demonstrated higher unadjusted observed diagnostic accuracy than planar bone scintigraphy (87.8% vs. 73.1%; *p* = 0.016), with higher observed sensitivity (79.2% vs. 53.3%), specificity (91.4% vs. 82.5%), positive predictive value (79.2% vs. 59.3%) and negative predictive value (91.4% vs. 78.8%). However, on a lesion-based analysis, whole-body bone SPECT/CT exhibited a low sensitivity of 47.5%, with particularly low detection rates for spine and pelvic metastases (29.4%), despite showing higher observed sensitivity than planar bone scintigraphy (20.1%). **Conclusions**: In two separate retrospective cohorts of SCLC patients, whole-body bone SPECT/CT showed higher unadjusted observed patients-based diagnostic performance than planar bone scintigraphy. However, its lesion-level sensitivity remained low, particularly in the spine and pelvis.

## 1. Introduction

Small cell lung cancer (SCLC) is a high-grade neuroendocrine carcinoma that accounts for 10–15% of all lung cancer cases [1]. Using the Veterans Administration Lung Cancer Study Group staging system, SCLC is classified into two stages: limited disease (confined to the ipsilateral hemithorax) and extensive disease (beyond the ipsilateral hemithorax) [2]. Owing to its highly aggressive features and early development of distant metastasis, approximately 70% of SCLC cases are classified as extensive disease at the initial staging work-up [1,2]. Limited disease is considered potentially curable when treated with a combination of systemic chemotherapy and local therapy, including radiotherapy and surgery [1,3]. Notably, in a recent clinical trial, the addition of durvalumab adjuvant therapy substantially improved overall survival among patients with limited disease, from 33.4 months to 55.9 months [4]. In contrast, despite combining chemotherapy with immunotherapy, patients with extensive disease still have a poor prognosis, with a 5-year overall survival rate of 12% [1,2,3]. The bone is one of the most common metastatic sites of SCLC, with a prevalence of 23–38% [5,6]. SCLC patients with bone metastasis have a worse prognosis, with a median overall survival of only 4.1 months, than those with non-osseous distant metastases [7]. Furthermore, patients with bone metastases are at risk of serious skeletal-related events, including pain, spinal cord compression, and pathological fractures, which can seriously affect their quality of life [5]. Therefore, the evaluation of bone metastasis is generally recommended as part of the SCLC staging work-up [3].

Owing to its high sensitivity and ability to assess the entire skeletal system in a single scan, bone scintigraphy using ^99m^Tc-labelled diphosphonates has been widely used to evaluate diverse skeletal diseases, including malignant bone diseases [8,9]. In cases of suspected bone metastasis without other metastases in SCLC, the European Society for Medical Oncology guideline recommends bone scintigraphy [3]. However, planar bone scintigraphy using a conventional dual-head gamma camera has inherent limitations related to detector geometry and two-dimensional image acquisition, which can contribute to a considerable proportion of indeterminate findings and thereby compromise diagnostic accuracy [10,11]. Over the last decade, cadmium–zinc–telluride (CZT)-based digital single-photon emission computed tomography/computed tomography (SPECT/CT) system employing full-ring 360° detector geometries has been used in clinical practice [12]. By enabling very close proximity of detectors to the patient, this new SPECT/CT system improves image sensitivity and resolution while reducing the whole-body SPECT/CT scanning time from 40–50 min to less than 20 min [8,13,14]. Consequently, this SPECT/CT system has improved the accessibility for imaging examinations and enhanced the diagnostic performance for detecting bone metastases [8]. However, to date, the diagnostic performance of whole-body 360° CZT bone SPECT/CT in detecting bone metastasis in patients with SCLC has not been reported.

Hence, in this study, we aimed to compare the diagnostic performance of planar bone scintigraphy using a conventional dual-head gamma camera and whole-body bone SPECT/CT using a full-ring 360° CZT camera for the detection of bone metastasis in patients with SCLC.

## 2. Materials and Methods

### 2.1. Patients

We retrospectively reviewed the electronic medical records of patients with histopathologically confirmed SCLC at our medical center between January 2021 and August 2025. Among them, 201 patients who underwent bone scintigraphy for staging work-up, performed as either planar bone scintigraphy using a conventional dual-head gamma camera or whole-body bone SPECT/CT using a full-ring 360° CZT camera, were included. Both modalities were routinely available and utilized within the same calendar period: the allocation of each modality was not randomized, but was determined based on physician discretion, patient preference, and camera availability. We excluded 26 patients who (1) had a history of another primary malignant disease (*n* = 3), (2) received any treatment before bone scintigraphy (*n* = 4), or (3) exhibited abnormal findings on bone scintigraphy (12 with planar bone scintigraphy and 7 with bone SPECT/CT) that required further clarification but did not undergo additional imaging examinations, such as ^18^F-fluorodeoxyglucose (FDG) positron emission tomography/CT (PET/CT) or magnetic resonance imaging (MRI) and clinical follow-up in our medical center, thereby precluding the classification of bone scintigraphy results (*n* = 19). All of these 19 patients were transferred to other institutions or lost to follow-up after the index examination, leaving no accessible medical records, confirmatory imaging, or treatment follow-up. A total of 175 patients with SCLC, comprising 93 who underwent planar bone scintigraphy and 82 who underwent whole-body bone SPECT/CT, were finally enrolled in the analysis.

### 2.2. Planar Bone Scintigraphy and Whole-Body Bone SPECT/CT Imaging Protocol

Planar bone scintigraphy and whole-body bone SPECT/CT were performed using a dual-head gamma camera (Infinia GP, GE Healthcare, Milwaukee, WI, USA) and a full-ring 360° CZT hybrid SPECT/CT system (VERITON-CT, Spectrum Dynamics Medical, Caesarea, Israel), respectively. The CZT SPECT/CT system comprised 12 CZT detector columns arranged at 360° and a 16-row CT detector. Both planar and SPECT/CT images were acquired 3–4 h after the intravenous injection of approximately 740 MBq ^99m^Tc-methylene diphosphonate (^99m^Tc-MDP).

For the planar bone scintigraphy, a continuous acquisition mode at a scanning speed of 12 cm/min was used with a low-energy general-purpose collimator (GE Healthcare, Milwaukee, WI, USA). The 20% symmetric window was centered at 140 keV. Anterior and posterior whole-body images were obtained for all patients, and additional spot images were acquired when necessary.

For whole-body bone SPECT/CT, low-dose CT for attenuation correction was initially performed at 100 kV and 30 mA. Afterwards, a whole-body SPECT scan was conducted in 6–7 bed positions with 100 s for each bed position. The number of bed positions per patient was determined based on individual patient height to ensure complete anatomical coverage. Photopeak energy windows were set as 140 keV with the 20% symmetric window. SPECT images were reconstructed using 3D ordered-subset expectation maximization with 4 iterations and 4 subsets. The reconstruction matrix size was 256 × 256, yielding an isotropic voxel size of 2.46 mm. CT-based attenuation correction was applied, while scatter correction was not performed. Image filtering included an intra-iteration filter convolution of 0.2 and a post-processing median filter with a kernel size of 3. Partial-volume correction was performed by incorporating CT-derived anatomical bone segmentation as a structural prior during SPECT reconstruction. For quantitative analysis, SPECT calibration was established using a uniform cylindrical phantom filled with a known activity concentration of ^99m^Tc verified via a calibrated dose calibrator, deriving a calibration factor of 1.074. Standardized uptake value was calculated using body weight normalization.

### 2.3. Image Analysis

All bone scintigraphy images were retrospectively and independently reviewed by two experienced nuclear medicine physicians (reader 1 with 3 years of clinical experience and reader 2 with 6 years of clinical experience in full-ring 360° CZT SPECT/CT interpretation) using MIM software version 7.3.7 (MIM Software Inc., Cleveland, OH, USA). The reviewers were aware of the patients’ age, sex, and diagnosis of SCLC, but were blinded to other clinical information and the results of other imaging studies. Each reviewer classified each detected bone lesion on a 3-point scale: 1, benign; 2, equivocal; and 3, malignant. For planar bone scintigraphy, all bone lesions with increased ^99m^Tc-MDP uptake were assessed with using the intensity, location, and pattern of uptake. For whole-body bone SPECT/CT, all bone lesions showing focally increased ^99m^Tc-MDP uptake on SPECT images were evaluated with concurrent consideration of CT findings [15]. Even in the absence of abnormal CT findings, lesions with abnormally increased ^99m^Tc-MDP uptake were considered malignant bone lesions. Conversely, lesions with abnormally increased ^99m^Tc-MDP uptake on SPECT were categorized as benign bone lesions, if the CT findings suggested benign etiologies, such as degenerative changes, post-traumatic changes, or bone islands. For lesion-level evaluation, skeletal lesions were spatially matched according to anatomical bone segments. To maintain a fully paired dataset for inter-reader agreement without selection bias, if a potential lesion in a designated anatomical segment was detected by only one reader, it was assigned a score of 0 (negative) by the other reader for that specific site. Inter-reader agreement was calculated using these initial, unassisted independent assessments from both readers prior to any consensus review. Afterwards, a joint consensus review was performed to resolve discrepancies and all evaluated bone lesions were categorized into two groups, benign and malignant, to reflect a real-world clinical setting and for comparison with the reference standard. For quantitative analysis, a spherical-shaped volume-of-interest was manually drawn over all bone lesions with increased radiotracer uptake, and the maximum standardized uptake value (SUVmax) of the lesions were measured.

### 2.4. Reference Standard

The reference standard was adjudicated by a dedicated panel consisting of one experienced nuclear medicine physician and two pulmonologists specializing in lung cancer, who were blinded to the initial planar scintigraphy and whole-body bone SPECT/CT scan interpretations. The final reference standard for bone metastasis was established on a per-patient and per-lesion basis through a composite diagnostic framework incorporating histopathology, concurrent FDG PET/CT and MRI images, clinical history, and clinical and radiological follow-up (median follow-up duration: 13.6 months; range: 6.0–66.2 months). All reference standard data were evaluated by independent panel members who were blinded to the initial planar scintigraphy and whole-body SPECT/CT study interpretations. A patient or lesion was defined as positive for bone metastasis if confirmed by either (1) histopathological verification via bone biopsy or (2) presence of characteristic metastatic features on baseline FDG PET/CT and/or MRI, accompanied by corresponding anatomical/functional treatment responses (for instance, osteoblastic sclerosis, decreased FDG uptake, or lesion regression) during serial follow-up imaging. A patient or lesion was defined as negative for bone metastasis if confirmed by either (1) absence of bone metastasis on initial FDG PET/CT and/or MRI, with sustained absence of bone metastasis during serial follow-up, or (2) negative findings on initial bone scintigraphy that remained persistently negative without radiological evidence of bone metastasis during follow-up. A lesion was defined as benign if initial FDG PET/CT and/or MRI demonstrated benign bone conditions, such as degenerative changes, post-traumatic change, or benign bone tumor, with sustained absence of bone metastasis during follow-up. The time interval between the index bone scintigraphy and reference imaging (FDG PET/CT or MRI) was strictly restricted to within 1 month, as all patients were undergoing initial baseline staging prior to definitive therapy. For two patients presenting with equivocal findings on reference imaging studies (one in planar bone scintigraphy cohort and one in bone SPECT/CT cohort), intervening treatment history was actively incorporated into the final adjudication. A lesion or patient was adjudicated as positive for bone metastasis, if chemotherapy or radiation therapy was initiated based on a clinical decision to treat the lesion as a bone metastasis, and subsequent serial follow-up imaging demonstrated a corresponding therapeutic response.

### 2.5. Statistical Analysis

Based on the reference standard, the diagnostic performance of planar bone scintigraphy and whole-body bone SPECT/CT was assessed using both patient- and lesion-based methods. Patients with diffuse bone metastases were included in only the patient-based analysis, but were excluded from the lesion-based analysis because of the innumerable lesions [16]. The main analytical outcome of this study was the evaluation and comparison of patient-based overall diagnostic accuracy between planar bone scintigraphy and whole-body bone SPECT/CT. All other patient- and lesion-based metrics were secondary and considered exploratory analyses. Patient-level diagnostic performance metrics, including sensitivity, specificity, positive predictive value (PPV), negative predictive value (NPV), and overall accuracy and their corresponding 95% confidence intervals (CIs) were calculated using the Clopper–Pearson exact method. To assess the potential impact of incorporation bias, a sensitivity analysis was performed for patient-based diagnostic performance after excluding patients whose negative status was adjudicated based solely on negative index bone scans with follow-up imaging studies. For a lesion-based analysis, inter-reader agreement between the two readers were firstly evaluated using linearly weighted kappa statistics. Afterwards, lesion-level diagnostic estimates (sensitivity and PPV) were evaluated using generalized estimating equations (GEEs) with a binary logit link function to account for the intra-patient correlation among multiple lesions within the same individual. An exchangeable working correlation structure was assumed, and robust (sandwich) standard error estimators were utilized to construct 95% confidence intervals and calculate *p*-values. The term ‘adjusted’ in the lesion-level results refers specifically to this adjustment for within-patient lesion clustering. For sensitivity analysis, patient clusters comprised patients with at least one metastatic bone lesion according to the reference standard, resulting in 26 patients in the planar scintigraphy cohort and 22 patients in the whole-body bone SPECT/CT cohort. For PPV analysis, patient clusters comprised patients with at least one lesion classified as positive on the respective imaging modality (true-positive or false-positive lesion), resulting in 24 patients in the planar scintigraphy cohort and 23 patients in the whole-body bone SPECT/CT cohort. After estimating the adjusted sensitivity and PPV for planar bone scintigraphy and whole-body bone SPECT/CT using the GEE models, the between-cohort differences were calculated on the probability scale within the corresponding GEE model, along with their 95% CIs and *p*-values. For raw diagnostic performance estimates, differences in diagnostic performance between the two independent cohorts were evaluated using the two-proportion comparison test (N-1 Chi-square test and Fisher’s exact test). In addition, detection rates according to the site of bone metastasis were ascertained. The parameters were compared using the Mann–Whitney U test, chi-square test, and Fisher’s exact test. For quantitative analysis, the SUVmax was compared among benign bone lesions, true-positive metastatic bone lesions, and false-positive lesions in the whole-body bone SPECT/CT group using the Kruskal–Wallis test. The value of SUVmax for discriminating metastatic bone lesions from false-positive and benign uptake sites among visually discernible lesions was assessed based on the area under the receiver operating characteristic (ROC) curve (AUC) value. To account for multiple lesions originating from the same patient, cluster-adjusted non-parametric methods were applied to derive the AUC and its 95% CIs. The lesions-based analysis using GEE models was performed using R software version 4.2.3 (R Foundation for Statistical Computing, Vienna, Austria) and all other statistical analyses were performed using MedCalc Statistical Software version 23.5.5 (MedCalc Software Ltd., Ostend, Belgium). A *p*-value of <0.05 was considered statistically significant for all tests.

## 3. Results

### 3.1. Comparison of Clinical Characteristics in Patients

The clinical characteristics of the planar bone scintigraphy (*n* = 93) and whole-body bone SPECT/CT (*n* = 82) patient groups are presented in Table 1. There were no significant differences in age, sex, tumor stage, or reference standard method between the two patient groups (*p* > 0.05). Furthermore, the prevalence of bone metastases was comparable between the two groups (32.3% in the planar bone scintigraphy group vs. 29.3% in the whole-body bone SPECT/CT group; *p* = 0.670).

### 3.2. Patient-Based Analysis

**Figure 1:**
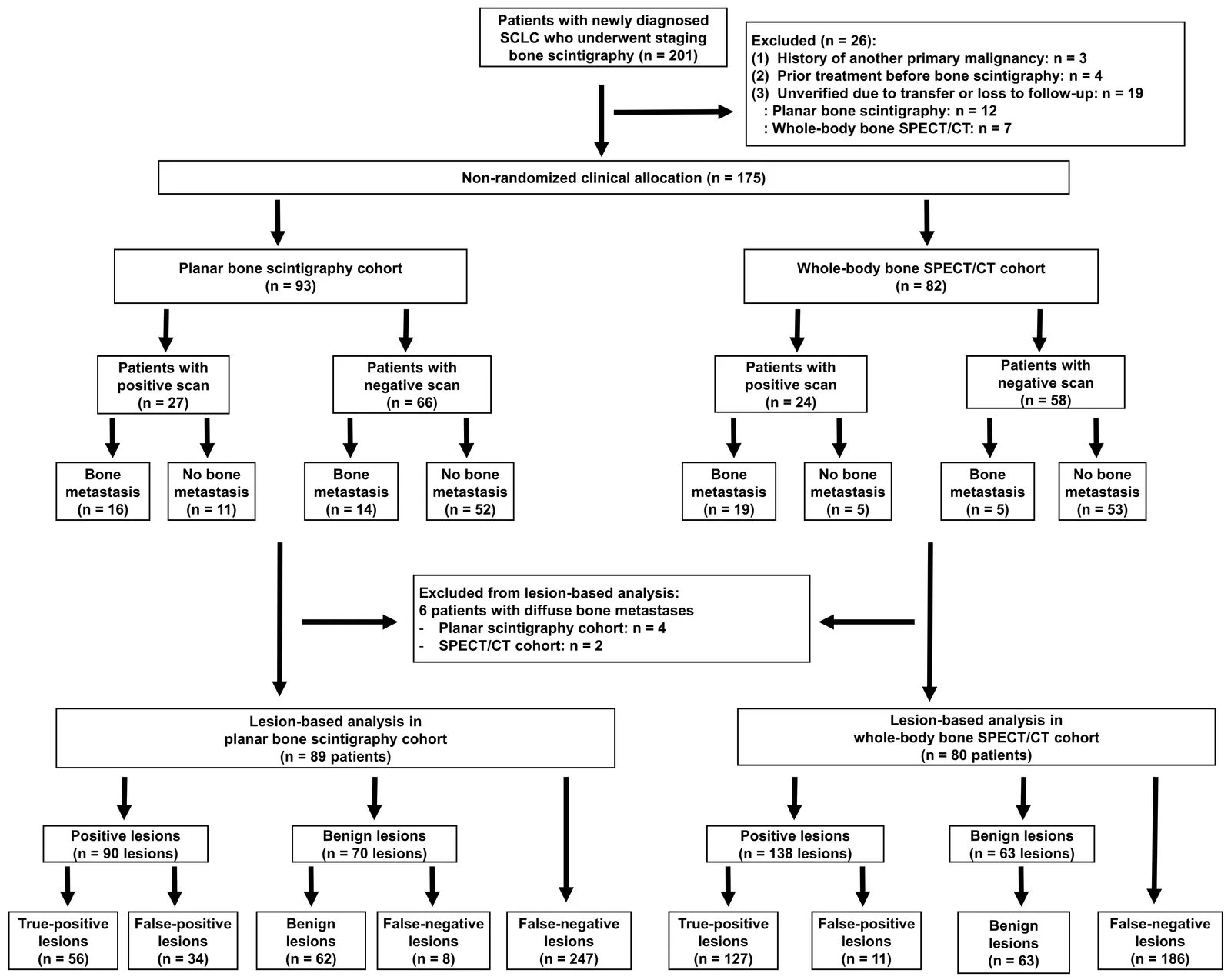
Flow diagram showing the process of analysis.

A schematic workflow of the participants is shown in Figure 1. Of the 93 patients, planar bone scintigraphy correctly diagnosed bone metastasis in 68 patients, including 16 of 30 patients with bone metastases and 52 of 63 patients without bone metastases (Table 2). On the other hand, 72 of 82 patients were correctly diagnosed using whole-body bone SPECT/CT, including 19 of 24 patients with bone metastases and 53 of 58 patients without bone metastases (Table 2).

**Figure 2:**
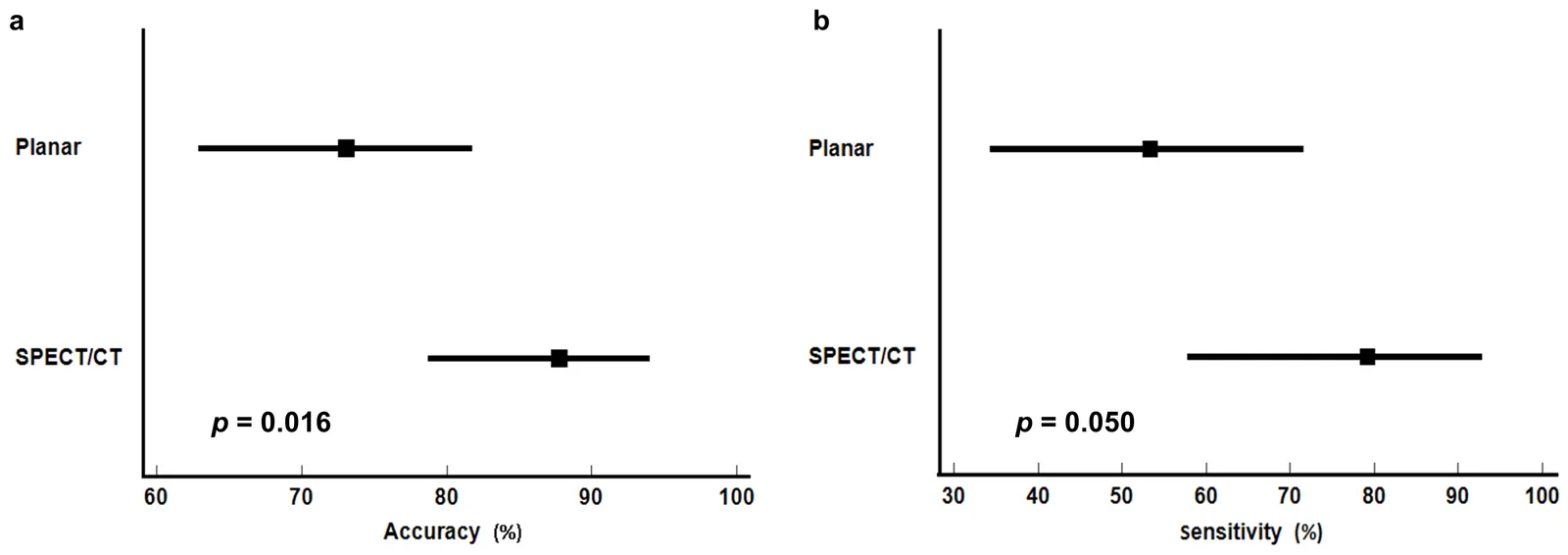
Forest plots for diagnostic accuracy (**a**) and sensitivity (**b**) of planar bone scintigraphy (planar) and whole-body bone SPECT/CT (SPECT/CT) on a patient-based analysis.

Based on these results, Table 3 summarizes the diagnostic performance of planar bone scintigraphy and whole-body bone SPECT/CT for detecting bone metastasis on a patient-based analysis. In the primary patient-based analysis, whole-body bone SPECT/CT showed significantly higher unadjusted observed overall diagnostic accuracy compared with planar bone scintigraphy (87.8% vs. 73.1%; difference, 14.7% [95% CI, 2.8–25.9%]; *p* = 0.016) (Figure 2a). In the exploratory analyses of secondary diagnostic parameters, whole-body bone SPECT/CT revealed significantly higher observed sensitivity (79.2% vs. 53.3%; difference, 25.9% [95% CI, 0.3–46.6]; *p* = 0.050) (Figure 2b). Observed specificity (91.4% vs. 82.5%), PPV (79.2% vs. 59.3%), and NPV (91.4% vs. 78.8%) were also higher in the whole-body bone SPECT/CT cohort, although these secondary parameters did not reach statistical significance (*p* > 0.05).

In a diagnostic performance analysis excluding patients with negative bone scintigraphy results whose reference standard relied solely on follow-up imaging examinations (3 in the planar cohort and 2 in the SPECT/CT cohort), the diagnostic performance metrics remained materially stable. In the planar cohort (*n* = 90), observed specificity, NPV, and accuracy were 81.7% (49 out of 60 patients; 95% CI, 69.6–90.5%), 77.8% (49 out of 63 patients; 95% CI, 70.1–83.4%), and 72.2% (65 out of 90 patients; 95% CI, 67.8–81.2%), respectively. In the SPECT/CT cohort (*n* = 80), observed specificity, NPV, and accuracy were 91.1% (51 out of 56 patients; 95% CI, 80.4–97.0%), 91.1% (51 out of 56 patients; 95% CI, 82.3–95.7%), and 87.5% (70 out of 80 patients; 95% CI, 78.2–93.8%), respectively. Consistent with the primary analysis, whole-body bone SPECT/CT maintained a significantly higher observed overall accuracy (difference, 15.3%; 95% CI, 3.1–26.8%; *p* = 0.014) and no significant difference in specificity (difference, 9.4%; 95% CI, −1.2–19.7%; *p* = 0.078). Notably, the difference in NPV reached statistical significance in this analysis (difference, 13.3%; 95% CI, 2.2–23.9%; *p* = 0.018).

### 3.3. Inter-Reader Agreement for Lesion-Based Analysis

Because of diffuse bone metastases, four patients in the planar bone scintigraphy group and two patients in the whole-body bone SPECT/CT group were excluded from the lesion-based analysis. Therefore, planar bone scintigraphy images from 89 patients and whole-body bone SPECT/CT image from 80 patients were included in the lesion-based analysis. The results of the lesion-level image evaluations by the two independent readers for planar bone scintigraphy and whole-body bone SPECT/CT are summarized in Table 4 and Table 5, respectively. For planar bone scintigraphy, a total of 160 bone lesions were found, of which 19.4% and 20.0% were classified as equivocal by reader 1 and reader 2, respectively. The inter-reader agreement for planar bone scintigraphy was moderate, with a linearly weighted kappa value of 0.492 (95% CI, 0.380–0.604). In contrast, among the 201 bone lesions found on whole-body bone SPECT/CT, only 6.5% and 6.0% were classified as equivocal by reader 1 and reader 2, respectively. The inter-reader agreement for whole-body bone SPECT/CT was almost perfect, with a linearly weighted kappa value of 0.847 (95% CI, 0.786–0.908).

Following joint consensus adjudication, the 160 bone lesions evaluated on planar bone scintigraphy were classified as 90 malignant lesions (56.3%) and 70 benign lesions (43.7%), whereas the 201 bone lesions evaluated on whole-body bone SPECT/CT were classified as 138 malignant lesions (68.7%) and 63 benign lesions (31.3%).

### 3.4. Lesion-Based Analysis

**Figure 3:**
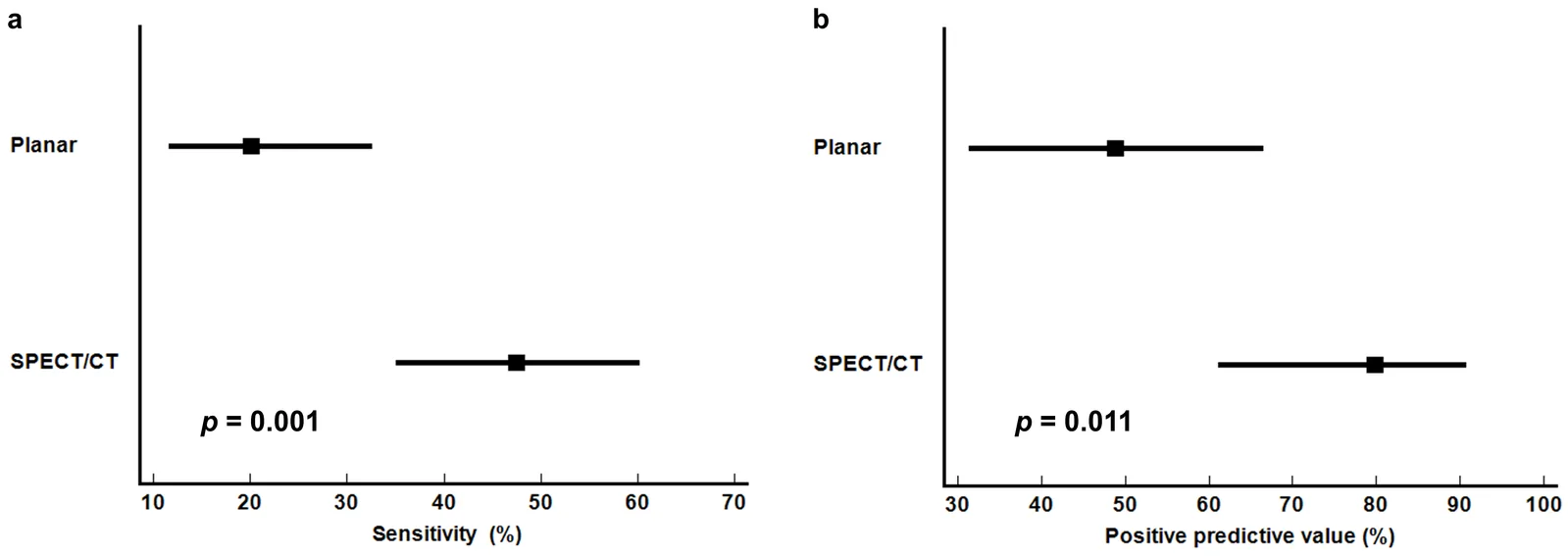
Forest plots for sensitivity (**a**) and positive predictive value (**b**) of planar bone scintigraphy (planar) and whole-body bone SPECT/CT (SPECT/CT) on a lesion-based analysis.

Based on the reference standard, 311 metastatic bone lesions were identified among the 89 patients with planar bone scintigraphy and 313 metastatic bone lesions among the 80 patients with whole-body bone SPECT/CT. Among the 90 lesions classified as malignant on planar bone scintigraphy by consensus reading, 56 were confirmed as true-positive metastatic bone lesions and 34 were false-positive bone lesions. Furthermore, among the 70 lesions classified benign on planar bone scintigraphy, 8 were confirmed as false-negative metastatic bone lesions. Meanwhile, among the 138 lesions classified as malignant on whole-body bone SPECT/CT by consensus reading, 127 were true-positive metastatic bone lesions and 11 were false-positives. All 186 false-negative metastatic bone lesions were completely undetected lesions on whole-body bone SPECT/CT. The sensitivity and PPV of the two imaging modalities on the lesion-based analysis were estimated using the GEE to address within-patient correlations among multiple lesions (Table 6). Whole-body bone SPECT/CT demonstrated higher observed sensitivity (47.5% vs. 20.1%; difference, 27.4% [95% CI, 10.8–44.0%]; *p* = 0.001) and PPV (79.8% vs. 48.8%; difference, 31.0% [95% CI, 7.3–54.6%]; *p* = 0.011) compared with planar bone scintigraphy (Figure 3).

**Figure 4:**
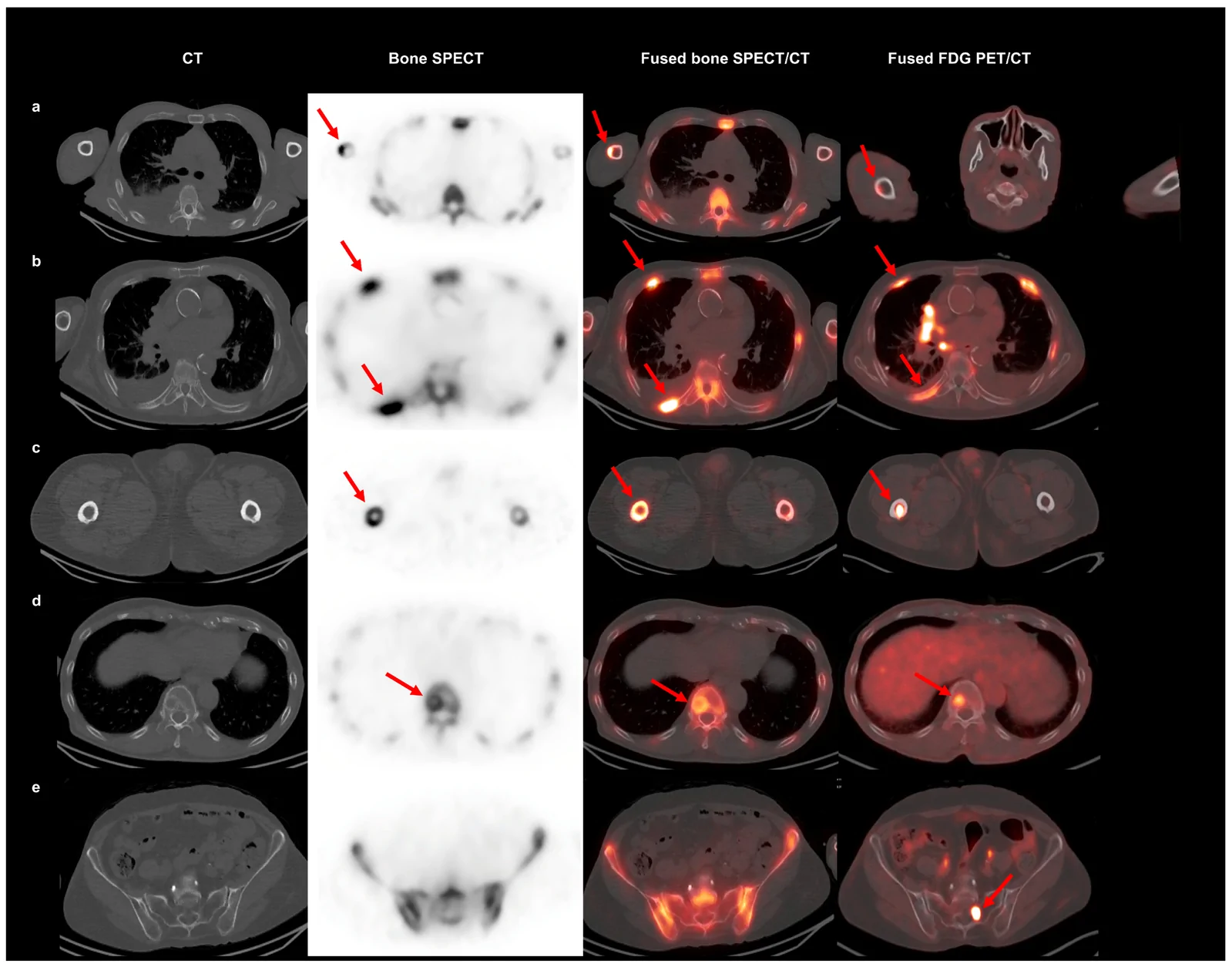
Representative cases of metastatic bone lesions on humerus (**a**), rib (**b**), femur (**c**), spine (**d**), and sacrum (**e**) on CT, bone SPECT, fused bone SPECT/CT, and fused ^18^F- fluorodeoxyglucose (FDG) PET/CT images. (**a**) On bone SPECT/CT images, focally increased ^99m^Tc-MDP uptake (SUVmax of 9.17) with no abnormal finding on CT images is observed in right humerus, while the lesion exhibited only mild FDG uptake (SUVmax of 3.52). (**b**) Intensely increased ^99m^Tc-MDP uptake with osteosclerotic change was seen in right 4th and 8th ribs (arrows) (SUVmax of 19.14 and 22.73 respectively) on bone SPECT/CT images, whereas relatively lower FDG uptake was demonstrated in these lesions (SUVmax of 8.59 and 6.68 respectively). (**c**) Bone SPECT/CT images revealed increased ^99m^Tc-MDP uptake (SUVmax of 6.72) along the cortex of right femur with relatively decreased uptake in the medulla. In contrast, focal intensely increased FDG uptake (SUVmax of 12.21) was seen in the medulla of the right femur. (**d**) The T11 vertebral body lesion demonstrated a centrally osteolytic component with peripheral osteosclerotic changes along its margin on CT image. Bone SPECT/CT showed increased ^99m^Tc-MDP uptake (SUVmax of 4.72) along the osteosclerotic portion, whereas FDG PET/CT exhibited increased FDG uptake (SUVmax of 4.50) in the central component of the lesion. (**e**) On CT and bone SPECT/CT images, no abnormal findings were observed in the sacrum. However, focal intensely increased FDG uptake (SUVmax of 17.44) was observed in the sacrum on FDG PET/CT image.

In addition to the overall sensitivity, we exploratory assessed the detection rates of planar bone scintigraphy and whole-body bone SPECT/CT according to the site of bone metastasis (Table 7). Whole-body bone SPECT/CT revealed relatively high detection rates of metastatic bone lesions in the skull and extremities (66.7%) and thoracic cage (51.3%) (Figure 4a–c). Similarly, planar bone scintigraphy also showed detection rates of 27.9% for lesions in the thoracic cage, which were higher than the adjusted overall sensitivity (20.1%). In contrast, both imaging modalities exhibited low detection rates for metastatic bone lesions in the spine and pelvis (29.4% for whole-body bone SPECT/CT and 15.0% for planar bone scintigraphy) (Figure 4d,e).

### 3.5. Quantitative Analysis for Whole-Body Bone SPECT/CT

**Figure 5:**
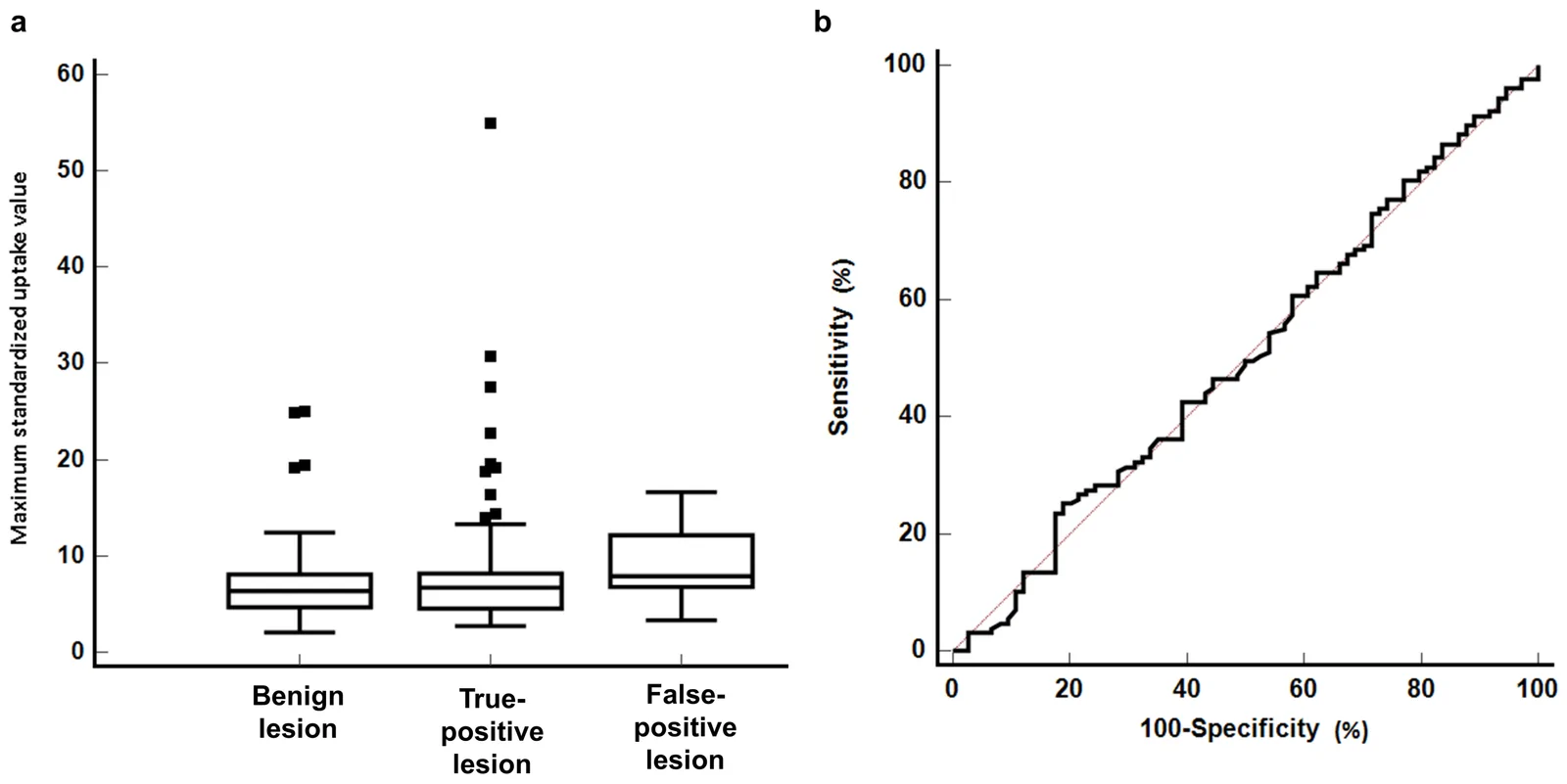
(**a**) Distribution of SUVmax of 63 benign, 127 true-positive, and 11 false-positive bone lesions. (**b**) The ROC curve of SUVmax for detecting bone metastasis among true-positive, false-positive, and benign bone lesions. The black line indicates the ROC curve, and the red diagonal line indicates the reference line (AUC = 0.5).

In the quantitative analysis, we evaluated whether the SUVmax on whole-body bone SPECT/CT could differentiate 127 true-positive metastatic bone lesions from 11 false-positive and 63 benign bone lesions. On the Kruskal–Wallis test, there was no significant differences in SUVmax among true-positive lesions (median, 6.72; interquartile range, 2.72–8.19), false-positive lesions (median, 7.90; interquartile range, 6.79–12.16), and benign bone lesions (median, 6.37; interquartile range, 4.67–8.10) (*p* = 0.135) (Figure 5a). On ROC curve analysis, the AUC value of SUVmax for discriminating bone metastases from false-positive and benign bone lesions was 0.503 (95% confidence interval [CI], 0.432–0.588) (Figure 5b).

## 4. Discussion

Despite the recommendation of bone scintigraphy for staging work-up in the guidelines, there are only a small number of studies on the diagnostic performance of bone scintigraphy for detecting bone metastasis in patients with SCLC [16,17,18]. All previous studies used planar bone scintigraphy [16,17,18], and only one study stated that additional SPECT was performed when necessary [17]. Of these studies, two showed low sensitivities of 37% and 61% but high specificities of 92% and 96%, respectively [16,17], whereas another study demonstrated a moderate sensitivity of 75% but low specificity of 58% [18]. All these studies suggest that planar bone scintigraphy plays an insufficient role in the diagnosis of SCLC bone metastasis, mainly because of its limited sensitivity [16,17,18]. In accordance with these results, our study showed a low sensitivity of 53.3% on a patient-based analysis for planar bone scintigraphy. The low sensitivity of planar bone scintigraphy is thought to result from the development of SCLC bone metastases [16]. In the literature, it has been thought that SCLC cells initially engraft within the bone marrow and, as they grow, progressively invade and destroy the surrounding cortical bone structure [16,19]. Because the accumulation of radiotracers on bone scintigraphy predominantly relies on an osteoblastic response to cancer cells, bone scintigraphy is less sensitive in detecting metastatic lesions confined to the bone marrow or associated with a minimal reactive response in the surrounding bone tissue [16,20]. In clinical studies, 64–88% of new lesions detected in a bone scintigraphy were preceded by adjacent metastatic lesions in the bone marrow observed in FDG PET/CT and MRI, suggesting that bone scintigraphy might have suboptimal diagnostic performance in detecting early-stage bone metastasis of SCLC [16,19].

In a previous meta-analysis study including 11 studies with diverse malignancies, mostly performed with a conventional dual-head SPECT/CT, bone SPECT/CT demonstrated superior diagnostic performance with less equivocal results than planar bone scintigraphy [21]. A Full-ring 360° CZT SPECT/CT system was found to feature substantially improved physical count sensitivity and spatial resolution compared with conventional SPECT/CT systems [22,23]. Recently, a prospective study directly comparing whole-body 360° CZT SPECT/CT with conventional Anger-camera in a diverse cancer population demonstrated comparable diagnostic agreement for detecting bone metastases while significantly reducing acquisition time [24]. Expanding on this emerging evidence, our study evaluated the diagnostic performance of a full-ring 360° CZT SPECT/CT system specifically in patients with SCLC. In line with the previous literature, our visual analyses demonstrated that whole-body bone SPECT/CT showed substantially lower proportion of equivocal findings (from 19.4% and 20.0% down to 6.5% and 6.0%, respectively) and achieved almost perfect inter-reader agreement, with a kappa value of 0.847 [21,25]. These findings indicate lower observed rates of equivocal classification and higher inter-reader reproducibility in the SPECT/CT cohort. However, because reader agreement was evaluated across different patient and lesion populations, these results should be interpreted as observed differences in reader agreement rather than conclusive proof that SPECT/CT resolves diagnostic uncertainty. In the primary patient-based analysis, the whole-body bone SPECT/CT cohort exhibited significantly higher unadjusted observed diagnostic accuracy (87.8% vs. 73.1%), alongside higher observed estimates for sensitivity, specificity, PPV, and NPV compared with the independent planar bone scintigraphy cohort. While planar bone scintigraphy historically tends to show adequate performance in either sensitivity or specificity, but rarely both [16,17,18], the whole-body bone SPECT/CT workflow achieved a more balanced performance profile, maintaining high specificity (91.4%) and NPV (91.4%) with reasonable sensitivity (79.2%). It should be noted that, because our study compared conventional planar scintigraphy directly with full-ring 360° CZT SPECT/CT, we could not isolate the independent effect of the full-ring 360° CZT camera geometry alone from the additive diagnostic benefit of the integrated SPECT/CT system itself. Furthermore, given the non-randomized allocation driven by physician discretion and camera availability, baseline exchangeability cannot be assumed, and our findings reflect unadjusted observed performance differences between independent cohorts rather than a pure modality effect. These methodological constraints should be considered when interpreting our results. Nevertheless, our findings suggest that whole-body bone SPECT/CT using a full-ring 360° CZT system demonstrated higher observed patient-based diagnostic accuracy estimates and fewer indeterminate findings during the initial bone metastasis evaluation of SCLC.

On a lesion-based analysis, whole-body bone SPECT/CT still maintained higher observed sensitivity and PPV compared with planar bone scintigraphy. However, despite a high PPV of 79.8%, whole-body bone SPECT/CT exhibited a low sensitivity of 47.5%. These results suggest that, although whole-body bone SPECT/CT could have a diagnostic role in a patient-based basis, its capability for precise lesion-by-lesion mapping of metastatic bone lesions remained constrained, necessitating complementary imaging modalities, such as FDG PET/CT or MRI, when detailed anatomical extension is required. In the exploratory site-specific analysis, whole-body bone SPECT/CT demonstrated detection rates >50.0% for bone lesions in the skull and extremities (66.7%), and thoracic cage (51.3%), whereas a lower detection rates of 29.4% was observed in the spine and pelvis. The spine, sacrum, and pelvic bone are known to exhibit relatively high normal physiological uptake and a higher incidence of degenerative changes. In a previous study that measured SUVmax of normal bone sites using a full-ring 360° CZT SPECT/CT in patients with prostate cancer, the spine and iliac bone revealed SUVmax of up to 6.00, with median SUVmax values of 2.78–3.26, whereas the median SUVmax of the skull, humerus, and femur was 1.29–1.99 [8]. Furthermore, in previous studies enrolling patients with various types of cancer, bone scintigraphy demonstrated relatively low detection rates for lesions in the spine, sacrum, and pelvis, mainly due to degenerative changes as well as increased normal background uptake [26,27]. Therefore, the relatively high physiological uptake and frequent degenerative changes in the spine and pelvis may obscure the uptake by small metastatic bone lesions, leading to reduced sensitivity. In addition, using a PET/CT-anchored reference standard likely contributed to a mechanical depression of the calculated lesion-based sensitivity in our study, since FDG PET/CT identifies early marrow-only metastases that inherently lack the osteoblastic turnover required for bone scintigraphy radiotracer uptake. Our findings delineate an observed performance profile of whole-body bone SPECT/CT in SCLC, characterized by higher patient-based diagnostic estimates but limited lesion-level sensitivity, particularly in the spine and pelvis. Because downstream clinical management, stage migration, and patient survival outcomes were not evaluated, these results remain restricted to diagnostic performance characteristics and failure patterns without confirming clinical utility.

One of the main advantages of bone SPECT/CT over planar bone scintigraphy is that it enables quantitative analysis [28]. Quantitative parameters derived from bone SPECT/CT, such as SUVmax, objectively assess the degree of radiotracer uptake by bone lesions, which can aid in the diagnosis of bone metastasis [8,28]. Notably, in prostate cancer, characterized by a high prevalence of osteoblastic bone metastasis, SUVmax has shown high diagnostic performance for detecting bone metastases, with reported AUC values of 0.874–0.949 [8,29,30]. For lung cancer, SUVmax also exhibited a high AUC value of 0.910 in a study enrolling 115 patients with adenocarcinoma, revealing a sensitivity of 87.7% and specificity of 80.7% with a cut-off value of 11.10 [15]. In contrast, another study that enrolled 22 SCLC patients with 119 non-small cell lung cancer patients found that a model incorporating diverse quantitative imaging parameters derived from bone SPECT images had the lowest AUC value (0.686–0.731) for detecting bone metastasis among diverse radiomic and clinical models [31]. In the current study, given the substantial proportion of false-negative lesions, assessing the overall diagnostic performance of SUVmax for detecting metastatic bone lesions might have limited utility. Nevertheless, we tried to evaluate whether SUVmax could effectively differentiate true metastatic lesions from other bone lesions among visually positive bone lesions. Our results exhibited that there were no significant differences in SUVmax between metastatic bone lesions, false-positive bone lesions, and benign bone lesions. Moreover, SUVmax showed an AUC value of 0.503 for discriminating bone metastasis from other visually positive bone lesions, which is too low to support its clinical utility. These may be explained by the bone marrow origin of SCLC bone metastasis and the relatively weak osteoblastic reactive response induced by cancer cells [16,19,20]. Hence, SUVmax alone might offer limited value for differentiating metastatic bone lesions from other bone lesions among visually discernible uptake sites in patients with SCLC. Consequently, diagnostic assessment should be performed by comprehensively considering lesion locations, uptake patterns, and CT findings. Our results contrast with those of previous studies on prostate cancer and non-small cell lung cancer, suggesting the restricted applicability of quantitative approaches to SCLC [8,15]. This underscores the need to evaluate the clinical utility of quantitative SPECT/CT analysis in a cancer-specific manner [15,32].

The present study had some limitations. First, this study was conducted retrospectively at a single medical center, requiring further studies to validate our results. Second, the diagnostic performances of planar bone scintigraphy and whole-body bone SPECT/CT have not been evaluated in the same patient population. Although there were no significant differences in clinical characteristics between the planar bone scintigraphy and whole-body bone SPECT/CT patient groups, this precluded a direct head-to-head comparison and may have affected the robustness of the results. Third, due to the retrospective design, we did not assess downstream clinical outcomes, stage migration, or treatment alterations; thus, this study cannot establish the clinical utility or efficacy of whole-body SPECT/CT as a decision-altering clinical tool. Fourth, the vast majority of reference standard diagnoses relied on composite imaging (FDG PET/CT and MRI) and clinical follow-up, with histopathological verification available in only a small subset of patients. Although comprehensive clinical and radiological follow-up was incorporated, the reliance on non-histological imaging endpoints may introduce potential lesion misclassification, particularly for early bone marrow-only metastases or atypical benign skeletal lesions [8,15]. Finally, differential verification bias cannot be fully excluded. While all of the patients with positive or indeterminate index scans underwent concurrent reference imaging, a subset of patients with completely negative bone scintigraphy results did not receive further advanced imaging and were verified predominantly through clinical and radiological follow-up. This differential work-up strategy carries the risk of under-detecting small, asymptomatic, or early marrow-confined metastatic lesions, which might lead to an overestimation of negative predictive value or diagnostic accuracy.

## 5. Conclusions

In the current study, whole-body bone SPECT/CT demonstrated higher unadjusted observed patient-based diagnostic performance, lower observed equivocal classification rates, and higher inter-reader agreement compared with planar bone scintigraphy in two separate, non-randomized retrospective cohorts of patients with SCLC evaluated for initial staging. However, because modality selection was non-randomized and clinical outcomes, stage migration, and management changes were not evaluated, our findings merely reflected observed diagnostic performance metrics rather than confirmed clinical utility. Furthermore, whole-body bone SPECT/CT exhibited low overall lesion-level sensitivity (47.5%), with marked limitations in the spine and pelvis (29.4%), highlighting the need for cautious interpretation when evaluating individual bone lesions.

## Figures and Tables

**Table 1:** Comparison of clinical characteristics in patient groups of planar bone scintigraphy and whole-body bone SPECT/CT.

Characteristic		Planar Bone Scintigraphy Group (*n* = 93)	Whole-Body Bone SPECT/CT Group (*n* = 82)	*p*-Value
Age (years) *		73 (38–85)	70 (37–90)	0.115
Sex	Men	82 (88.2%)	71 (86.6%)	0.753
	Women	11 (11.8%)	11 (13.4%)	
T stage	T1–T2	28 (30.1%)	28 (34.1%)	0.569
	T3–T4	65 (69.9%)	54 (65.9%)	
N stage	N0	14 (15.1%)	17 (20.7%)	0.441
	N1–N2	37 (39.8%)	26 (31.7%)	
	N3	42 (45.2%)	39 (47.6%)	
M stage	M0	31 (33.3%)	29 (35.4%)	0.778
	M1	62 (66.7%)	53 (64.6%)	
VALSG system	Limited disease	31 (33.3%)	26 (31.7%)	0.819
	Extensive disease	62 (66.7%)	56 (68.3%)	
Bone metastasis	Absence	63 (67.7%)	58 (70.7%)	0.670
	Presence	30 (32.3%)	24 (29.3%)	
Number of metastatic bone lesions in patients with bone metastases *		6 (1–46)	3 (1–66)	0.992
Reference standard method	Histopathological confirmation	4 (4.3%)	3 (3.7%)	0.928
	Composite imaging with follow-up imaging studies	86 (92.5%)	77 (93.9%)	
	Imaging follow-up only	3 (3.2%)	2 (2.4%)	

* Data are the median (range); VALSG, Veterans Administration Lung Study Group.

**Table 2:** Results of a patient-based analysis of the performance of planar bone scintigraphy and whole-body bone SPECT/CT in detecting bone metastasis.

Modality		Final Diagnosis	
		Bone Metastasis	No Bone Metastasis
Planar bone scintigraphy (*n* = 93)	Positive	16	11
	Negative	14	52
	Total	30	63
Whole-body bone SPECT/CT (*n* = 82)	Positive	19	5
	Negative	5	53
	Total	24	58

**Table 3:** Patient-based analyses of the diagnostic performance of planar bone scintigraphy and whole-body bone SPECT/CT in detecting bone metastasis.

Parameter	Planar Bone Scintigraphy	Whole-Body Bone SPECT/CT	Absolute Difference	*p*-Value
Sensitivity (%)	53.3 (34.3–71.7)	79.2 (57.8–92.9)	25.9 (0.3–46.6)	0.050
Specificity (%)	82.5 (70.9–90.9)	91.4 (81.0–97.1)	8.9 (−3.6–21.1)	0.153
Positive predictive value (%)	59.3 (43.6–73.3)	79.2 (57.8–92.9)	19.9 (−5.6–41.8)	0.130
Negative predictive value (%)	78.8 (71.4–84.7)	91.4 (81.0–97.1)	12.6 (−0.3–24.9)	0.053
Accuracy (%)	73.1 (62.9–81.8)	87.8 (78.7–94.0)	14.7 (2.8–25.9)	0.016

Numbers in parentheses are the 95% confidence intervals.

**Table 4:** Distribution of lesion-level diagnostic classifications by two independent readers and final consensus reading of planar bone scintigraphy.

		Reader 1				
		Negative	Benign	Equivocal	Malignant	Total
Reader 2	Negative	0	1	1	0	2 (1.3%)
	Benign	3	36	11	10	60 (37.5%)
	Equivocal	1	8	15	8	32 (20.0%)
	Malignant	0	11	4	51	66 (41.3%)
	Total	4 (2.5%)	56 (35.0%)	31 (19.4%)	69 (43.1%)	160 (100.0%)
`Final consensus reading			70 (43.7%)	-	90 (56.3%)	160 (100.0%)

**Table 5:** Distribution of lesion-level diagnostic classifications by two independent readers and final consensus reading of whole-body bone SPECT/CT.

		Reader 1				
		Negative	Benign	Equivocal	Malignant	Total
Reader 2	Negative	0	5	2	0	7 (3.5%)
	Benign	1	48	1	2	52 (25.9%)
	Equivocal	1	1	6	4	12 (6.0%)
	Malignant	0	1	4	125	130 (64.7%)
	Total	2 (1.0%)	55 (27.4%)	13 (6.5%)	131 (65.2%)	201 (100.0%)
Final consensus reading			63 (31.3%)	-	138 (68.7%)	201 (100.0%)

**Table 6:** Lesion-based analyses of the diagnostic performance of planar bone scintigraphy and whole-body bone SPECT/CT in detecting bone metastasis.

Parameter	Planar Bone Scintigraphy	Whole-Body Bone SPECT/CT	Absolute Difference	*p*-Value
Sensitivity (%)				
Raw	18.0 (13.9–22.7)	40.6 (35.1–46.2)	22.6 (15.6–29.3)	<0.001
Adjusted *	20.1 (11.6–32.6)	47.5 (35.1–60.3)	27.4 (10.8–44.0)	0.001
Positive predictive value (%)				
Raw	62.2 (51.4–72.2)	92.0 (86.2–96.0)	29.8 (18.9–40.7)	<0.001
Adjusted *	48.8 (31.3–66.6)	79.8 (61.2–90.8)	31.0 (7.3–54.6)	0.011

* Adjusted values were estimated using generalized estimating equations with an exchangeable working correlation structure and robust (sandwich) standard errors to account for within-patient correlation among multiple lesions. Numbers in parentheses are the 95% confidence intervals.

**Table 7:** Detection rates of planar bone scintigraphy and whole-body bone SPECT/CT according to the site of bone metastasis.

Anatomical Site	Planar Bone Scintigraphy	Whole-Body Bone SPECT/CT
Spine & pelvis	15.0% [10.0–21.6%] (29/193)	29.4% [21.8–38.8%] (50/170)
Spine	14.3% (14/98)	31.5% (29/92)
Sacrum	4.8% (1/21)	8.7% (2/23)
Pelvic bones	18.9% (14/74)	34.5% (19/55)
Thoracic cage	27.9% [17.9–41.5%] (24/86)	51.3% [39.2–65.9%] (61/119)
Rib	23.8% (15/63)	53.3% (49/92)
Sternum	37.5% (3/8)	33.3% (3/9)
Scapula	50.0% (6/12)	56.2% (9/16)
Clavicle	0.0% (0/3)	0.0% (0/2)
Skull & extremities	9.4% [1.9–27.4%] (3/32)	66.7% [38.1–100.0%] (16/24)
Skull	0.0% (0/4)	66.7% (2/3)
Humerus	14.3% (1/7)	55.6% (5/9)
Femur	9.5% (2/21)	75.0% (9/12)
Total	18.0% [13.9–22.7%] (56/311)	40.6% [35.1–46.2%] (127/313)

Numbers in parentheses are (the number of true positive on images/the total number of metastatic bone lesions). Numbers in brackets are 95% confidence intervals.

## Data Availability

The datasets presented in this study are available on request from the corresponding author due to ethical restrictions.

## Abbreviations

The following abbreviations are used in this manuscript:

AUC	Area under the receiver operating characteristics curve
CI	Confidence interval
CZT	Cadmium–zinc–telluride
FDG	^18^F-fluorodeoxyglucose
GEE	Generalized estimating equations
MRI	Magnetic resonance imaging
NPV	Negative predictive value
PET/CT	Positron emission tomography/computed tomography
PPV	Positive predictive value
ROC	Receiver operating characteristic
SCLC	Small cell lung cancer
SPECT/CT	Single-photon emission computed tomography/computed tomography
SUVmax	Maximum standardized uptake value
^99m^Tc-MDP	^99m^Tc-methylene diphosphonate
